# Yeast Mnn9 is both a priming glycosyltransferase and an allosteric activator of mannan biosynthesis

**DOI:** 10.1098/rsob.130022

**Published:** 2013-09

**Authors:** Alexander Striebeck, David A. Robinson, Alexander W. Schüttelkopf, Daan M. F. van Aalten

**Affiliations:** 1 Division of Molecular Microbiology, University of Dundee, Dundee DD1 5EH, UK; 2 Division of Biological Chemistry and Drug Discovery, University of Dundee, Dundee DD1 5EH, UK; 3 MRC Protein Phosphorylation and Ubiquitylation Unit, College of Life Sciences, University of Dundee, Dundee DD1 5EH, UK

**Keywords:** cell wall, glycobiology, glycosyltransferase, mannan, M-Pol I, protein crystallography

## Abstract

The fungal cell possesses an essential carbohydrate cell wall. The outer layer, mannan, is formed by mannoproteins carrying highly mannosylated *O*- and *N*-linked glycans. Yeast mannan biosynthesis is initiated by a Golgi-located complex (M-Pol I) of two GT-62 mannosyltransferases, Mnn9p and Van1p, that are conserved in fungal pathogens. *Saccharomyces cerevisiae* and *Candida albicans mnn9* knockouts show an aberrant cell wall and increased antibiotic sensitivity, suggesting the enzyme is a potential drug target. Here, we present the structure of *Sc*Mnn9 in complex with GDP and Mn^2+^, defining the fold and catalytic machinery of the GT-62 family. Compared with distantly related GT-78/GT-15 enzymes, *Sc*Mnn9 carries an unusual extension. Using a novel enzyme assay and site-directed mutagenesis, we identify conserved amino acids essential for *Sc*Mnn9 ‘priming’ α-1,6-mannosyltransferase activity. Strikingly, both the presence of the *Sc*Mnn9 protein and its product, but not *Sc*Mnn9 catalytic activity, are required to activate subsequent *Sc*Van1 processive α-1,6-mannosyltransferase activity in the M-Pol I complex. These results reveal the molecular basis of mannan synthesis and will aid development of inhibitors targeting this process.

## 2. Introduction

*N*-linked glycosylation is an abundant post-translational protein modification on secreted proteins in both bacteria and eukaryotes [1]. The synthesis of eukaryotic *N*-linked glycans is initiated at the luminal side of the endoplasmic reticulum (ER). Here, the core glycan GlcNAc_2_Man_9_Glc_3_ is transferred onto the asparagine in the sequon N–X–S/T. This core glycan is then trimmed by mannosidases and glucosidases, a process conserved across the eukaryotic kingdom and used as protein-folding quality control [2,3]. The glycosylated proteins are then transported to the Golgi apparatus, where numerous glycosyltransferases (GTs) extend the core glycan to form a rich, cell-type-specific diversity of *N*-linked glycosylated proteins. While the ER-localized part of the pathway is well conserved across most eukaryotes, the type and activity of the Golgi GTs is cell-type-specific [4–7]. In addition, differential regulation and protein acceptor specificity of these GTs also lead to different types of glycosylation of different proteins in the same cell. The precise activities, substrate specificities and molecular mechanisms for most of these GTs remain largely unknown.

**Figure 1.**
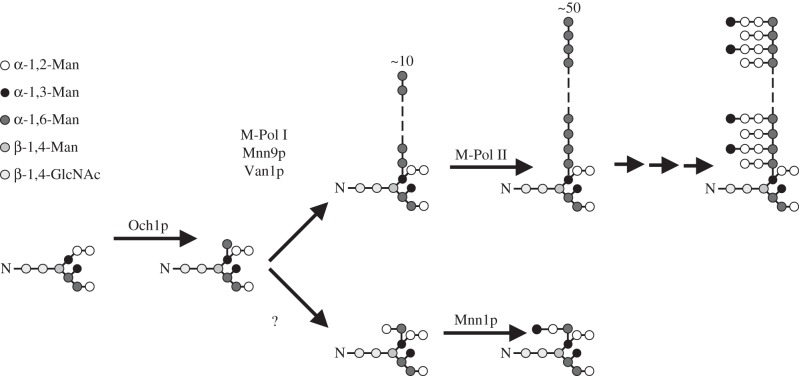
Diagram of mannosylation pathways in the yeast Golgi. *N*-linked glycosylated proteins arrive from the ER and are extended with a single α-1,6-linked mannose by Och1p. This residue either can be further elongated to a primary α-1,6-linked mannose backbone by Mnn9p and Van1p (M-Pol I, top) to form the mannan backbone on mannoproteins or is further extended by an unknown mannosyltransferase with an α-1,2-linked mannose that eventually leads to a core glycosylation structure for proteins that are retained in internal compartments (bottom).

The synthesis of *N*-linked glycans and their decoration in the Golgi apparatus has been extensively studied in the yeast *Saccharomyces cerevisiae*. Like many other yeasts and fungi, *S. cerevisiae* possesses a core cell wall structure of chitin and glucan, further decorated by elaborate glycans in the outer cell wall [8,9]. Mannose and phosphomannose derivatives are the only sugars added to glycans in the yeast Golgi, a process performed by mannosyltransferases that use GDP-mannose (GDP-Man) as the donor substrate [10,11]. Upon entry into the *cis*-Golgi, core *N*-linked glycans receive an α-1,6-linked mannose by the transferase Och1p [12,13] (figure 1). This residue is extended with an α-1,2 and α-1,3-linked mannose in proteins targeted for retention in cellular organelles [14]. While the α-1,2-mannosyltransferase remains unknown, Mnn1p has been identified as the GT-71 mannosyltransferase that attaches the terminal α-1,3-linked mannose [15]. The second class of glycosylated proteins in *S. cerevisiae* is composed of mannoproteins. These proteins form mannan, the outermost layer of the fungal cell wall that can make up to 40% of the cell dry weight [13,16,17]. Mannoproteins have been shown to carry up to 200 mannose residues on each of their *N*-linked glycans [18]. The synthesis of mannoproteins starts by the extension of the α-1,6-linked mannose to form a backbone of at least 10 α-1,6-linked mannose residues [16] (figure 1). This reaction is carried out by the M-Pol I heterodimeric complex, composed of the mannosyltransferases *Sc*Mnn9p and *Sc*Van1p [19]. This backbone is further extended with α-1,6-linked mannose residues by the heteropentameric mannosyltransferase complex M-Pol II [19–21] (figure 1). Additional mannosyltransferases add branches of α-1,2- or α-1,3-linked mannose and phosphomannose to this backbone [11,22]. Finally, mannoproteins leave the *trans-*Golgi in vesicles that fuse with the membrane and release their content to the extracellular space where the mannoproteins either associate with or are transglycosylated into β-glucan to form the outer layer of the fungal cell wall [8].

It remains unclear how the *S. cerevisiae* Golgi apparatus targets only *certain N*-linked glycans towards the synthesis of mannoproteins. Stolz & Munro [23] showed that an immunoprecipitated heterodimeric M-Pol I complex of active *Sc*Mnn9p and inactive *Sc*Van1p extends the pseudo-acceptor α-1,6-mannobiose with a single α-1,2-linked mannose *in vitro*, whereas an immunoprecipitated heterodimeric M-Pol I complex of inactive *Sc*Mnn9p and active *Sc*Van1p extends the same acceptor with several α-1,6-linked mannose residues. However, when a core glycosylated protein was used as the acceptor, a mannose backbone was only formed with an M-Pol I complex possessing active *Sc*Mnn9p and active *Sc*Van1p. The authors suggested that an *N*-glycosylated substrate protein arrives at the complex and interacts with *Sc*Mnn9p, which in turn changes its mannosyltransferase activity according to the type of acceptor protein. If the protein is to remain in the internal compartments, then *Sc*Mnn9p would attach an α-1,2-linked mannose to the α-1,6-linked mannose to prevent it from further elongation to a mannose backbone, whereas mannoproteins targeted for secretion would receive an α-1,6-linked mannose from *Sc*Mnn9p that would be extended by *Sc*Van1p to form the primary mannan backbone (figure 1). However, when *S. cerevisiae* Mnn9p and Van1p were coexpressed in *Pichia pastoris*, purified and used for an *in vitro* assay, the only observed reaction product was an α-1,6-linked mannose backbone attached to mannobiose [24]. It is possible that the immunoprecipitated samples used in the study by Stolz & Munro [23] were contaminated with an α-1,2-mannosyltransferase and that M-Pol I is solely active on mannoproteins. *In vitro* studies with the individually expressed components will help resolve this controversy, and aid in the dissection of the glycosyltransferase and activating functions of this pair of enzymes in the M-Pol I complex.

*Sc*Mnn9p and *Sc*Van1p are type II membrane proteins, possessing a short cytosolic *N*-terminal domain followed by a transmembrane domain that is required for anchoring to the Golgi membrane, but not for interaction of the two enzymes [20]. The transmembrane domain is followed by a linker and the C-terminal globular catalytic domain. *Sc*Mnn9p and *Sc*Van1p belong to the CAZy glycosyltransferase family GT-62. This family contains metal-dependent, retaining mannosyltransferases that are predicted to possess the GT-A fold [25] and use GDP-Man as their donor substrate. There is currently no structural information available for any GT-62 family member, limiting our understanding of the molecular details of the interaction between the M-Pol I enzymes and their acceptor proteins, as well as the catalytic mechanism.

Here, we report the structure of the mannosyltransferase domain of *Sc*Mnn9 in complex with manganese and GDP. The structure reveals that *Sc*Mnn9 possesses the GT-A fold and shows structural similarity to GT families 15 and 78. However, *Sc*Mnn9 possesses a unique extrusion that may act as a molecular ruler or multimerization domain. Using a novel fluorimetric coupled enzyme assay, we determine steady-state kinetics of wild-type *Sc*Mnn9 and identify conserved residues that are necessary for activity. Furthermore, we show that *Sc*Mnn9 and its product are necessary for processive M-Pol I mannosyltransferase activity *in vitro.* Using complementation studies in a yeast *ΔMNN9* strain, we demonstrate that the presence of inactive full-length *Sc*Mnn9 partially rescues the *ΔMNN9* phenotype*.* The results presented here show that both the presence and the priming activity of *Sc*Mnn9 are required for the formation of the α-1,6-mannose backbone of mannan proteins.

## 3. Results and discussion

### 3.1. *Sc*Mnn9 is structurally similar to GT-15 and GT-78 mannosyltransferases

**Figure 2.**
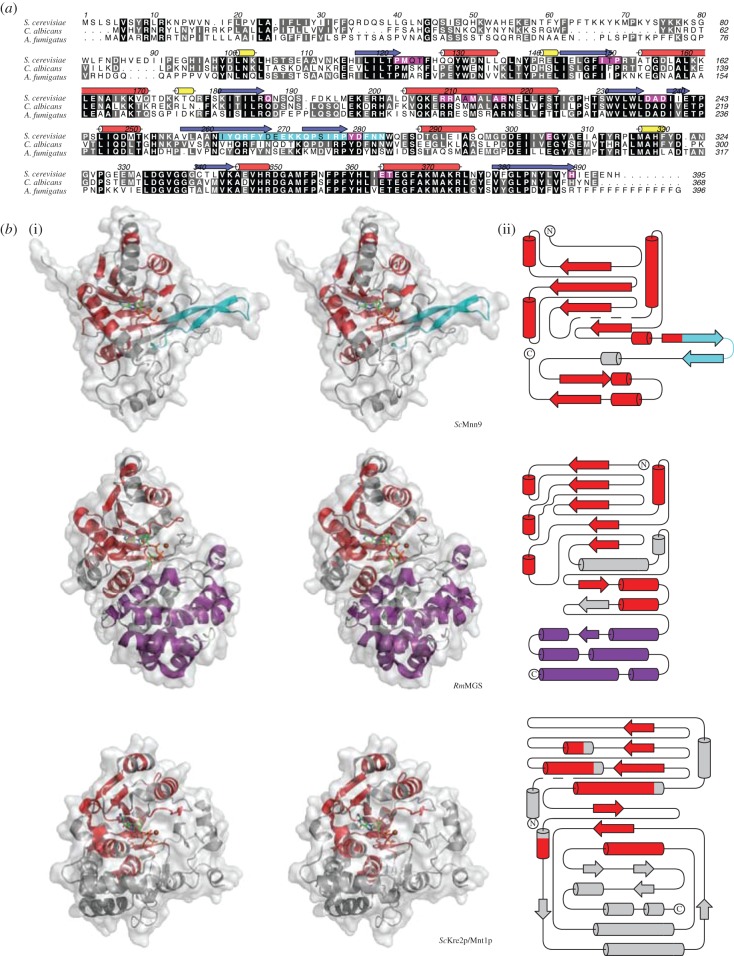
Structure and conservation of Mnn9. (*a*) Protein sequence alignment of Mnn9 proteins of fungal species. Red barrels indicate α-helices, blue arrows indicate β-strands. Black shading indicates completely conserved residues, grey shading indicates residues that are conserved between two of the three species. Purple shading indicates residues that form the active site of *Sc*Mnn9. The cyan box indicates the stretch that forms the extension in *Sc*Mnn9. Residue numbers are shown for *Sc*Mnn9. (*b*) (i) Stereoscopic cartoon/surface representation of the structures of *Sc*Mnn9 in complex with GDP and Mn^2+^, *Rm*MGS in complex with GDP-Man and Mg^2+^, and *Sc*Kre2p/Mnt1p in complex with GDP and Mn^2+^. Structural similarities between all three transferases are shown in red. Unique features are shown in cyan (hairpin loop of *Sc*Mnn9) and purple (dimerization domain of *Rm*MGS). GDP and GDP-Man are shown as sticks and metals are shown as brown spheres. (ii) Topology of *Sc*Mnn9, *Rm*MGS and *Sc*Kre2p/Mnt1p with the same colour codes as in the cartoon representations. Disordered regions of the proteins are shown as dashed lines.

**Figure 3.**
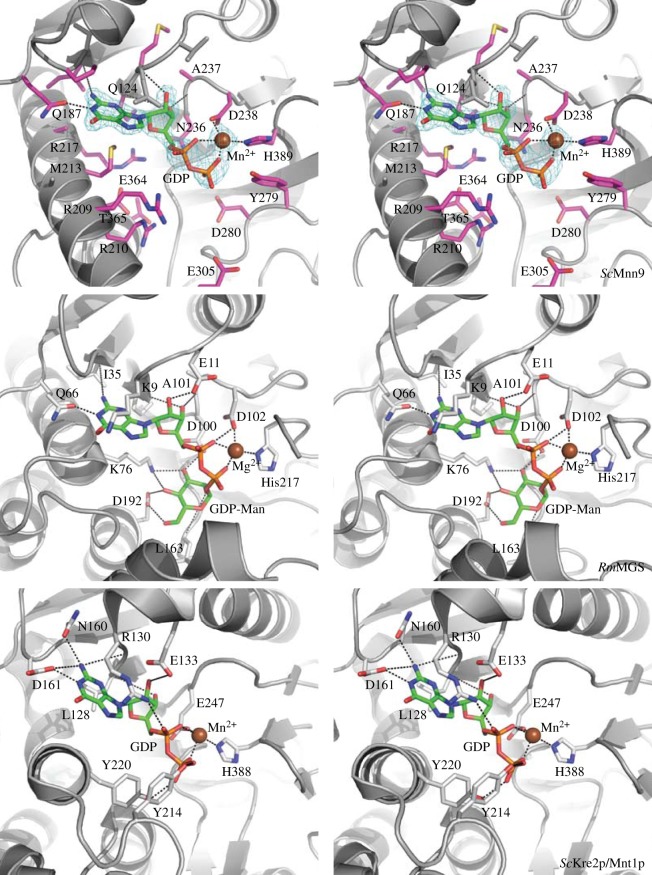
Active site of *Sc*Mnn9, *Rm*MGS and *Sc*Kre2p/Mnt1p. A close-up of the active sites of *Sc*Mnn9 and structurally related enzymes shown in figure 2*b. Sc*Mnn9 residues within 6 Å of GDP are shown as sticks with purple carbon atoms. The unbiased |*F*_O_|-|*F*_C_| map (1.75*σ*) is shown as cyan mesh around GDP and Mn^2+^ in *Sc*Mnn9. Mn^2+^ and Mg^2+^ are shown as brown spheres. Hydrogen bonds are shown as dashed black lines.

Mnn9 proteins from yeasts and filamentous fungi possess high levels of sequence conservation (figure 2*a*). To study the molecular basis of α-1,6-mannosyl transfer activity of Mnn9, we determined the crystal structure of the enzyme from *S. cerevisiae*. An *Escherichia coli* expression construct (*Sc*Mnn9) was created covering the glycosyltransferase core (93–395) only (figure 2*a*). Expression of this construct, followed by Ni–IMAC, anion exchange and size-exclusion chromatography, yielded 5 mg of pure protein sample per litre of bacterial culture. The recombinant protein was used to grow crystals from ammonium sulfate solutions. The *Sc*Mnn9 structure was solved by a 2.2 Å single-wavelength anomalous dispersion (SAD) phasing experiment with a mercury derivative and refined against 2.0 Å synchrotron diffraction data of a mutant (D236N) in the presumed GT-A DxD catalytic motif, in complex with GDP and Mn^2+^, yielding a final *R*/*R*_free_ of 0.19/0.24 (table 1). The structure reveals that *Sc*Mnn9, and by extension the entire GT-62 glycosyltransferase family, adopts the GT-A fold as previously proposed by sequence analysis [25] (figure 2*b*). Seven β-strands form a sheet that is covered on both sides by α-helices. Using the DALI server (http://ekhidna.biocenter.helsinki.fi/dali_server), the *Rhodothermus marinus* mannosylglycerate synthase from GT-76 (*Rm*MGS, PDB: 2Y4M; figure 2*b*) [26] as well as the *S. cerevisiae* α-1,2-mannosyltransferase *Sc*Kre2p/Mnt1p from GT-15 (PDB: 1S4O; figure 2*b*) [27] were identified as structural homologues (r.m.s.d. = 2.4 Å on 158 equivalent Cα atoms or r.m.s.d. = 3.4 Å on 167 equivalent Cα atoms, respectively). *Rm*MGS uses GDP-Man as the donor substrate to transfer mannose onto d-glycerate, d-lactate or glycolate [26]. *Sc*Kre2p/Mnt1p synthesizes *O*-linked oligomannose and the terminal oligomannose decorations on mannoproteins [22]. Superposition of *Sc*Mnn9, *Rm*MGS and *Sc*Kre2p/Mnt1p reveals the structural similarity derived from the GT-A fold around the catalytic site (figure 2*b*). However, *Sc*Mnn9 has a unique hairpin loop formed by two additional β-strands (I262-N283; figure 2*a,b*). This loop is positioned in line with the active site and could serve a number of purposes. In order to address this question, we replaced this loop with a tetraglycine stretch. However, this construct did not produce soluble protein in *E. coli*, suggesting that the protein lacking the hairpin loop is misfolded and/or degraded. The loop could potentially act as a molecular ruler for the formation of a mannose backbone of defined length or act as a guide to recognize and correctly position protein *N*-linked glycans for mannosyl transfer. By contrast, *Rm*MGS has a more extended C-terminus formed by six helices and a short β-strand, whereas *Sc*Kre2p/Mnt1p does not contain any protruding features. Interestingly, the interactions between GDP and the enzymes are very similar (figure 3). The guanine forms hydrogen bonds between the N1 amine and the amide oxygen of a glutamine (*Sc*Mnn9 and *Rm*MGS) or an aspartic acid (*Sc*Kre2p/Mnt1p). In all three enzymes, the guanine is buried by residues with relatively long side chains (i.e. Q124 in *Sc*Mnn9, K9 in *Rm*MGS and R130 in *Sc*Kre2p/Mnt1p; figure 3). The ribofuranose forms extensive hydrogen bonds with side-chain residues forming the active site. The metal is coordinated by a histidine side chain, a carboxylate and the pyrophosphate moiety of GDP. The histidine, common to many retaining GT-A GTs, occupies a similar position within the active site of all three transferases (figure 3) and is one of the very few (five) conserved side chains in the sequences of the GT-15, GT-62 and GT-76 GTs compared here (figure 3). The carboxylate metal ligand is part of the canonical GT-A fold DxD motif (figures 2*a* and 3). Both the α- and β-phosphates of the GDP interact with the metal, similar to the *Rm*MGS enzyme (figure 3).

**Table 1.** Details of data collection and structure refinement. Values between parentheses correspond to the highest resolution shell.

	*Sc*Mnn9 + Hg-SAD	*Sc*Mnn9-D236N + Mn^2+^ + GDP
space group	P6_5_22	P6_5_22
cell dimensions		
* a, b, c* (Å)	57.05, 57.05, 330.17	57.09, 57.09, 330.91
* α, β, γ* (°)	90, 90, 120	90, 90, 120
resolution range (Å)	25.0–2.2	55.0–2.0
number of observed reflections	1 342 417	366 234
number of unique reflections	19 947	23 775
redundancy	37.8 (33.4)	9.7 (10.1)
*I/*σ**(*I*)	37.0 (13.6)	11.8 (5.0)
completeness (%)	99.9 (100.0)	100.0 (100.0)
*R*_merge_	0.148 (0.579)	0.149 (0.482)
number of protein residues	288	288
number of water molecules	—	148
*R*_work_, *R*_free_	—	0.19, 0.24
r.m.s.d. from ideal geometry		
bond lengths (Å)	—	0.012
bond angles (°)	—	1.43
*B*-factors (Å^2^)		
protein	—	17.9
ligand	—	GDP: 34.7 Mn^2+^: 45.2
water	—	19.7

Despite the fact that *Sc*Mnn9-D236N was soaked or co-crystallized with GDP-Man, electron density for mannose was not observed. The mannose of GDP-Man in the *Rm*MGS complex forms hydrogen bonds with K76 and D192, and several backbone amides (figure 3). A mannose may be similarly coordinated in the *Sc*Mnn9 through the structurally equivalent R209 and E364/T365, respectively. Despite the striking similarities between *Sc*Mnn9 and *Sc*Kre2p/Mnt1p, the reactions catalysed of both GTs result in a different glycosidic bond. Owing to the lack of a binary GDP-Man complex structure of both GTs, we can speculate that only the positions of the side chains in the pocket around residues R209, R210, D280, E305 and T365 result in the formation of an α-1,6-glycosidic bond.

### 3.2. Recombinant *Sc*Mnn9 possesses mannosyltransferase activity *in vitro*

**Figure 4.**
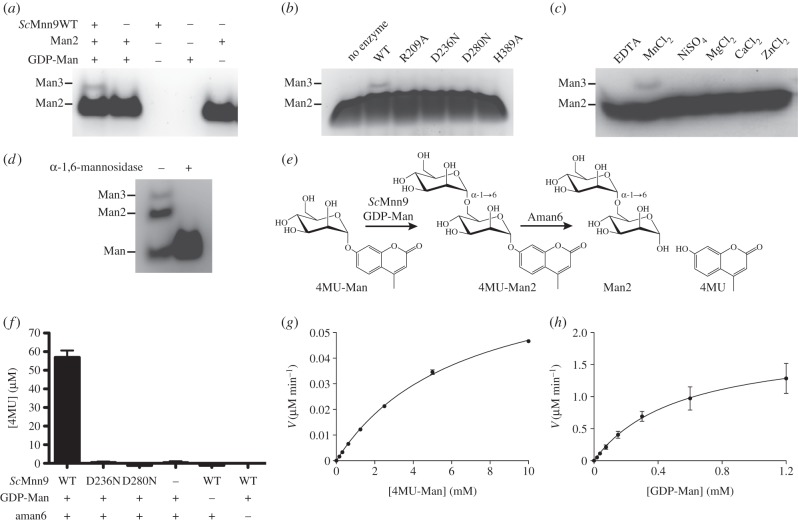
Activity assay of *Sc*Mnn9 wild-type and mutants. (*a*) FACE gel of the reaction products of *Sc*Mnn9 wild-type incubated with GDP-Man, α-1,6-mannobiose (Man2) and MnCl_2_ or controls. (*b*) Same as (*a*), but incubated with wild-type or mutants of *Sc*Mnn9. (*c*) Same as (*a*), but incubated with 10 mM of MnCl_2_, other divalent cations or EDTA. (*d*) FACE gel of a reaction containing *Sc*Mnn9, mannose, GDP-Man and MnCl_2_. The reaction products were either treated or not treated with an α-1,6-mannosidase. (*e*) Diagram of the chemical reaction that is the foundation for the coupled enzyme assay to determine steady-state kinetics for *Sc*Mnn9. 4MU-Man is extended by *Sc*Mnn9 to 4MU-Man2, which in turn acts as a minimal substrate for *B. circulans* Aman6, an α-1,6-mannosidase. The release of fluorescent 4MU was measured at an excitation wavelength of 360 nm and an emission wavelength of 460 nm. (*f*) Bar chart of the measured 4MU released after the reaction of *Sc*Mnn9 wild-type, D236N and D280N in presence and absence of GDP-Man and Aman6 mannosidase. Error bars indicate the standard error of the mean (s.e.m.), *n* = 3. (*g*,*h*) Steady-state kinetics of *Sc*Mnn9 (*g*) in the presence of GDP-Man (1.2 mM) and variable 4MU-Man concentrations or (*h*) in the presence of 4MU-Man (10 mM) and variable GDP-Man concentrations. Error bars indicate s.e.m., *n* = 3.

To date, enzyme activity of *Sc*Mnn9p has been demonstrated only in the presence of *Sc*Van1p [23,24]. We tested the activity of bacterially expressed *Sc*Mnn9 in the presence of manganese, GDP-Man, and the model acceptor substrates mannose and α-1,6-linked mannobiose. Reaction products were separated and observed by fluorophore-assisted carbohydrate gel electrophoresis (FACE; figure 4*a,d*), showing that *Sc*Mnn9 alone is able to transfer mannose from the sugar donor onto the acceptor substrates, forming mannotriose. We then investigated the role of conserved amino acid side chains in the active site (figures 2 and 4*b*). R209 lines the putative mannose binding site, and mutation to alanine results in the loss of *Sc*Mnn9 activity (figure 4*b*). Similar effects were reported for the equivalent K76A mutation in *Rm*MGS [26]. *Sc*Mnn9 D236 is the first aspartic acid in the canonical GT-A DxD catalytic motif, and mutation to the isosteric asparagine results in the loss of activity (figure 4*b*), similar to the previously reported less conservative D236A mutation [23]. The equivalent *Rm*MGS Asp100 forms a hydrogen bond with the mannose O3 hydroxyl (figure 2*b*). Although we were unable to obtain a complex of *Sc*Mnn9 in complex with an intact donor, inspection of the superimposed *Rm*MGS complex suggests that *Sc*Mnn9 Asp280 would also line the putative mannose binding site, positioned close to the O2 hydroxyl group. Mutation of this aspartic acid to asparagine (D280N) results in an inactive enzyme (figure 4*b*). *Sc*Mnn9 His389 coordinates the manganese and is indispensable for catalytic activity (figure 4*b*). Manganese is required for activity, and cannot be substituted by other divalent cations, such as magnesium or calcium (figure 4*c*). Interestingly, *Sc*Mnn9 was able to extend mannose to α-1,6-mannobiose and -mannotriose, as shown by treatment with a commercially available α-1,6-specific mannosidase (figure 4*d*).

To study *Sc*Mnn9 steady-state kinetics, we developed a novel coupled enzyme assay that involves only one additional enzyme, in contrast to the established glycosyltransferase assays where the release of GDP is measured by NADH oxidation through pyruvate kinase and lactate dehydrogenase [28]. Here, we used the gene product of *Bacillus subtilis* TN-31 *aman6* (Aman6), an α-1,6-mannosidase [29,30], as a coupling enzyme (figure 4*e*). This mannosidase has been reported to act on mannotriose as a minimal substrate [29]. We discovered that this enzyme also liberates 4-methylumbelliferone (4-MU) from 4MU-α-d-Man-(1 → 6)-d-Man (4MU-Man2), but crucially not from 4MU-Man. Thus, only in the presence of active *Sc*Mnn9, which transfers a mannose onto 4MU-Man, does the Aman6 mannosidase liberate fluorescent 4MU from the resultant 4MU-Man2 product (figure 4*f*).

This assay was used to establish steady-state kinetics for wild-type *Sc*Mnn9 (figure 4*g,h*). The *K*_M,app_ and *V*_max_ determined for the 4MU-Man acceptor are 6.5 (±0.3) mM and 77.7 (±2) nM min^−1^, respectively, resulting in a *k*_cat_ of 0.2 min^−1^. The *K*_M,app_ and *V*_max_ for GDP-Man are 0.5 (± 0.2) mM and 1.9 (±0.3) µM min^−1^, respectively, resulting in a *k*_cat_ of 3.8 min^−1^. Compared with the *K*_m_ and *V*_max_ values measured for *Rm*MGS using glycerate as an acceptor, *Sc*Mnn9 seems to have low affinity for both of its substrates [26,31]. Interestingly, the *k*_cat_ for *Sc*Mnn9 and GDP-Man is comparable to *k*_cat_ of *Rm*MGS and GDP-Man (1.9 min^−1^ [26]). By contrast, *Sc*Kre2p/Mnt1p is considerably faster than *Sc*Mnn9, with *k*_cat_ of 12.8 s^−1^ for GDP-Man and 10.8 s^−1^ for methyl-α-mannoside, whereas with a *K*_m_ of 26 mM, the acceptor analogue methyl-α-mannoside appears to be a poor substrate for *Sc*Kre2p/Mnt1p [27]. The low substrate affinity of *Sc*Mnn9 and *Sc*Kre2p/Mnt1p *in vitro* might be a result of the artificial acceptor substrates used. *Sc*Krep2/Mnt1p is not only involved in *O*-linked mannosylation of serines and threonines but also attaches α-1,2-linked mannose to the α-1,6-mannose backbone formed by M-Pol I and II [32–34]. Hence, it recognizes rather complex substrates. The substrate of *Sc*Mnn9 is an *N*-linked core glycan extended with a mannose attached by Och1p [19]—structurally rather dissimilar from the 4MU-Man pseudo-acceptor used here. Furthermore, *Sc*Mnn9p is found in the multimeric glycosyltransferase complexes M-Pol I and II [19,21], and intermolecular interactions in these complexes may well increase the affinity of *Sc*Mnn9p for its substrates. It is also possible that *Sc*Mnn9 has a comparatively low affinity for its substrates in order to limit its consumption of cellular GDP-Man, which may be particularly important as M-Pol I activity is the starting point of extensive additional mannosylation [21], requiring large amounts of additional GDP-Man.

### 3.3. *Sc*Mnn9 is required as a priming enzyme and an allosteric activator for *Sc*Van1 polymerase activity

**Figure 5.**
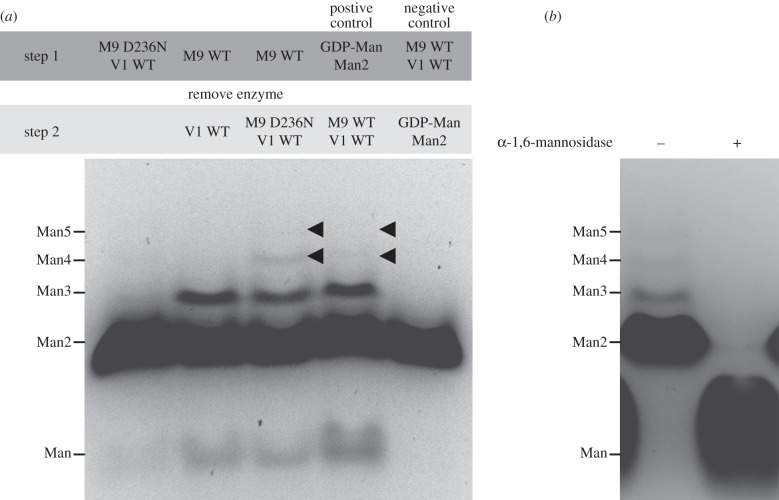
Synergy of *Sc*Mnn9 and *Sc*Van1. (*a*) FACE gel of ANTS-labelled reaction products of the reaction of *Sc*Mnn9 (M9), *Sc*Van1 (V1), GDP-Man, α-1,6-mannobiose (Man2) and MnCl_2_. First lane: *Sc*Mnn9-D236N and *Sc*Van1 wild-type were incubated together, the reaction was stopped and the products were labelled before the separation on a FACE gel. The faint band above Man2 is assumed to be leakage from lane 2 (noted on other gels, not shown). Second and third lanes: *Sc*Mnn9 WT was used in absence of *Sc*Van1; *Sc*Mnn9 WT was then removed using a 10 000 MWCO filter, and *Sc*Van1 WT alone (second lane) or *Sc*Mnn9-D236N and *Sc*Van1 WT (third lane) were added to the reaction. The second step of the reaction was stopped and the products were labelled before being separated on a FACE gel. Fourth lane: control in which the substrates were incubated in the absence of enzymes, spun through the filter and subsequently the reaction was carried out by addition of the enzymes. The products formed due to the presence of substrates and GTs. Fifth lane: control in which the enzymes were incubated in the absence of the substrates, removed by the filter and the filtrate was enriched with the substrates. Products did not form due to the absence of GTs. (*b*) *Sc*Mnn9, *Sc*Van1, GDP-Man, α-1,6-mannobiose and MnCl_2_ were incubated, and the reaction products were either treated (+) or not treated (–) with the *B. circulans* Aman6 α-1,6-mannosidase. The products after the reaction were labelled with ANTS and run on a FACE gel.

The formation of oligomannose by M-Pol I has been demonstrated *in vitro* [19,23,24]. However, it remains unclear how Mnn9p and Van1p act synergistically. In addition to recombinant *Sc*Mnn9, we were also able to separately express and purify the *Sc*Van1 glycosyltransferase domain (residues 87–535) using similar procedures as described for *Sc*Mnn9 earlier. In order to ensure that the truncations of *Sc*Mnn9 and *Sc*Van1 are still able to form a complex, we measured the interaction between the two proteins using biolayer interferometry, yielding a *K*_d_ of 480 nM (*k*_on_ 6.2 × 10^2^ M^−1^ s^−1^; *k*_off_ 3.2 × 10^−4^ s^−1^). We then tested whether *Sc*Mnn9 is required as a ‘priming’ α-1,6-mannosyltransferase and/or essential for extension of the α-1,6 mannan backbone with a polymerase-like activity. Recombinant *Sc*Van1 and GDP-Man were mixed either with the reaction product of *Sc*Mnn9 (α-1,6-mannotriose) alone or with the reaction product together with the catalytically inactive *Sc*Mnn9-D236N mutant. The reaction products were labelled with 8-aminonaphthalene-1,3,6-trisulfonic acid (ANTS) and observed on a FACE gel (figure 5*a*). The resultant oligomers were of α-1,6-linked mannose, as demonstrated by susceptibility to α-1,6-mannosidase treatment (figure 5*b*). Strikingly, *Sc*Mnn9-priming mannosyltransferase activity is required to allow *Sc*Van1 polymerase activity. However, it is the *Sc*Mnn9 protein, but not its catalytic activity, that is necessary and sufficient for this *Sc*Van1 polymerase activity, suggesting the involvement of an allosteric mechanism in the M-Pol I complex (figure 5*a*). This mechanism has been proposed previously using complexes immunoprecipitated from *S. cerevisiae* where either *Sc*Mnn9p or *Sc*Van1p were inactive [23]. However, the same report showed that *Sc*Van1p is able to form oligomannose without the product of *Sc*Mnn9p, a result we (and others [24]) were not able to reproduce with recombinant *Sc*Mnn9. Our data suggest that in the context of the M-Pol I complex, *Sc*Mnn9 acts as both a priming glycosyltransferase and an allosteric activator, whereas *Sc*Van1 is the polymerase synthesizing the α-1,6 mannose backbone on mannoproteins (figure 1). The formation of free mannose in lanes 2 and 3 of figure 5*a* is likely to be the result of GDP-Man hydrolysis (i.e. glycosyltransfer onto a water molecule acceptor).

### 3.4. *Sc*Mnn9p catalytic activity is indispensable for mannoprotein synthesis in yeast

**Figure 6.**
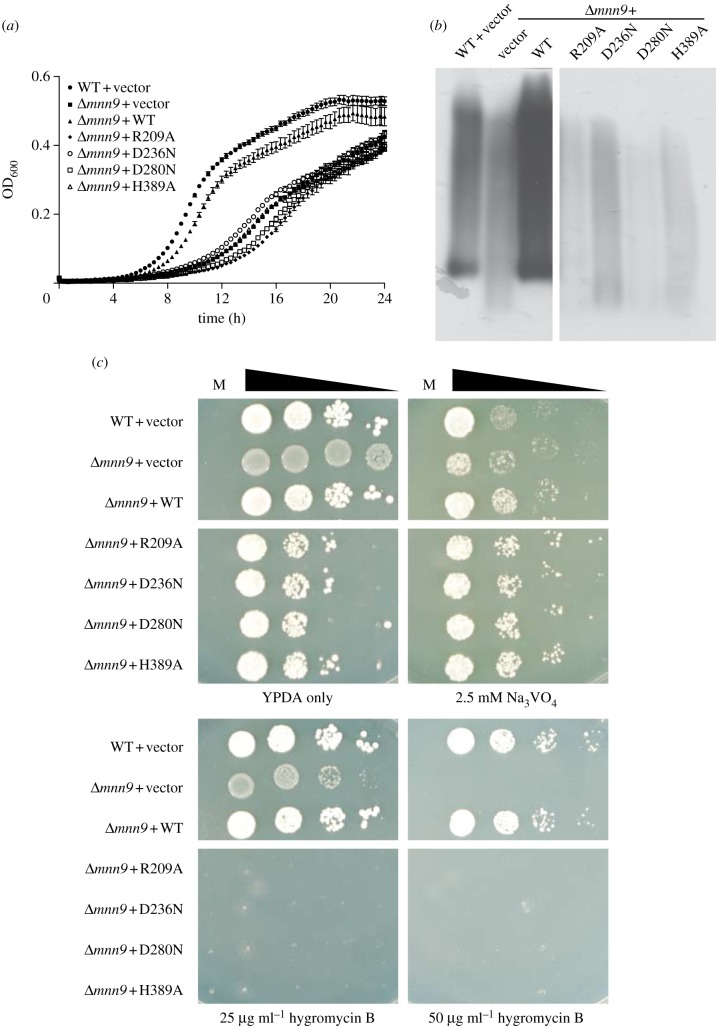
Phenotypic analysis of native and complemented *Saccharomyces cerevisiae* *ΔMNN9* strains. (*a*) Growth curve of *S. cerevisiae* BY4741 wild-type or *ΔMNN9* transformed with either pRS315 vector control (vector) or different mutations of the *MNN9* gene. Growth of *S. cerevisiae* was followed by measurement of absorption at 600 nm. Error bars indicate s.e.m., *n* = 3. (*b*) 2,3,5-triphenyltetrazolium-labelled PAGE gel to determine the state of glycosylation of extracellular invertase of the *S. cerevisiae* transformants indicated. A Coomassie brilliant blue-stained PAGE gel as loading control can be found in the electronic supplementary material, figure S1. (*c*) *Saccharomyces cerevisiae* BY4741 wild-type or *ΔMNN9* transformed with the same constructs as used in panel (*a*) grown on YPD plates either without any supplement or with 2.5 mM Na_3_VO_4_, or 25 and 50 µg ml^−1^ hygromycin B. Cells were spotted in a serial dilution on plates, M indicates the medium-only control.

Strains of *Saccharomyces cerevisiae* and *Candida albicans* with defects in mannan synthesis show hypersensitivity to hygromycin B and reduced sensitivity to sodium orthovanadate [35,36]. Guided by the crystal structure, we have identified catalytically inactive mutants of *Sc*Mnn9 (figure 4*b*) that can now be used to dissect the function of the protein with the help of a *ΔMNN9* strain of *S. cerevisiae*. Wild-type and point mutants R209A, D236N, D280N and H389A of the *MNN9* gene, including 5′- and 3′-UTR, were cloned into the yeast expression plasmid pRS315 [37]. *Saccharomyces cerevisiae* BY4741 wild-type and *ΔMNN9* cells were transformed with these plasmids. Successfully transformed cells were selected on DO-Leu(–) plates. *ΔMNN9* cells transformed with wild-type *MNN9* grew after a longer lag phase at a similar rate, but to slightly lower density, in YPD compared with wild-type cells carrying the empty pRS315 vector control (figure 6*a*). Similar observations were made in complementation experiments of the *C. albicans* *ΔMNN9* mutant [38]*.* Cells lacking *MNN9* or carrying *MNN9* with a point mutation grew notably slower and did not reach stationary phase after 24 h, in contrast to wild-type and reconstituted *MNN9* (figure 6*a*). Thus, cells expressing catalytically inactive *Sc*Mnn9p show growth kinetics comparable with those measured for cells lacking *MNN9.* Furthermore, wild-type cells and *ΔMNN9* cells carrying the wild-type *MNN9* showed similar glycosylation patterns of the extracellular invertase, whereas the *ΔMNN9* and cells containing the *MNN9* mutants showed very low glycosylation (figure 6*b*; electronic supplementary material, figure S1). This indicates that the point mutations in *MNN9* lead to the same effect as the deletion of *MNN9* in YPD. On YPD plates, the *ΔMNN9* phenotype is presented as an increased sensitivity to hygromycin B and reduced susceptibility to Na_3_VO_4_ (figure 6*b*). The susceptibility to Na_3_VO_4_ was not decreased in cells carrying the point mutations in *MNN9* compared with WT cells. (figure 6*b*). However, cells lacking *MNN9* or carrying the point mutants were susceptible to lower concentrations of hygromycin B than wild-type or reconstituted cells (figure 6*b*). Notably, cells expressing *Sc*Mnn9p with a point mutation did not grow at the lowest concentration of hygromycin B, whereas *MNN9* knockout cells were still able to grow, suggesting that the complete loss of *MNN9* may activate rescue pathways for cell survival, whereas these pathways are not being activated in the presence of inactive *Sc*Mnn9p. In contrast to the results obtained in the invertase assay (figure 6*b*), this result indicates that the presence of an inactive form of *Sc*Mnn9p has a more severe impact on cell wall architecture than the complete absence of the transferase. The difference between the two results could be explained by the additional pressure through the presence of hygromycin B that may activate additional rescue pathways. The results gathered from the *in vivo* experiments are similar to previous reports, although these only covered either complete knockouts or a single point mutant [23,38].

## 4. Concluding remarks

The *Sc*Mnn9 glycosyltransferase is essential for the formation of mannosylated proteins that form mannan in the yeast cell wall. The results presented here define the structure of this key enzyme, the first example of a structure of the GT-62 family of GTs. *Sc*Mnn9 adopts a GT-A fold with an unusual hairpin loop extension. Residues that form the active site and are conserved across fungal species are important for activity. Furthermore, we show that *Sc*Van1 is shifted into an active conformation only upon binding of *Sc*Mnn9 and requires the priming product of *Sc*Mnn9 for GT polymerase activity. However, it has to be noted that this report uses a non-physiological substrate and that the activities of *Sc*Mnn9 and *Sc*Van1 may well be different with native acceptors (i.e. *N*-glycosylated proteins). Nevertheless, these results indicate the importance of *Sc*Mnn9p for the formation of the mannan backbone. Further work is required to capture *Sc*Mnn9 in complex with *Sc*Van1 or in complex with a physiologically relevant substrate such as an *N*-linked glycosylated peptide or protein. Previous reports have suggested that knockouts of *MNN9* in *C. albicans* show phenotypes that are inconsistent with virulence, making *Sc*Mnn9p a potential drug target [38]. Future studies will exploit the data presented here to identify potent inhibitors that may serve as lead compounds for new therapeutics.

## 5. Material and methods

### 5.1. Cloning and protein expression

The genes encoding *Sc*Mnn9p and *Sc*Van1p lacking the first 92 amino acid residues (*Sc*Mnn9) or the first 86 amino acid residues (*Sc*Van1), excluding the N-terminal cytoplasmic domain, the transmembrane domain and the predicted disordered region, were cloned from genomic DNA of *S. cerevisiae* S288c into pNIFTY/maltose-binding protein (MBP), introducing a MBP, a hexahistidine stretch and a tobacco etch virus (TEV) protease recognition sequence at the N-terminus using the oligonucleotides 5′-AT**GGCGCC**GAAGGTCATATTGCACATTATGATTTGAACAAATTGC-3′ (forward) and 5′-AT**GGATCC**ATCAATGGTTCTCTTCCTCTATGTGATAAACC-3′ (reverse) for *Sc*Mnn9, and 5′-GC**GGCGCC**ATGGGCATTGGTGTATCCACGC-3′(forward) and 5′-AT**GGATCC**ATTACTCTGATTGTCTTCTCTTCTCTCTTTCCC (reverse) for *Sc*Van1 (restriction sites *Kas*I and *Bam*HI, respectively, shown in bold). A plasmid containing *Sc*Mnn9 wild-type was used to introduce different point mutations by site-directed mutagenesis. To do so, the following oligonucleotides were used: R209, 5′-GCTTTAGATGTTCAAAAGGAAGCTCGTGCAGC AATGGCTTTGGCG-3′ and 5′-CGCCAAAGCCATTGCTGCACGAGCTTCCTTTTGAACATCTAAAGC-3′; D236N, 5′-GGTGCTGTGGCTAAATGCCGATATTATAGAGACACC-3′ and 5′-GGTGTCTCTATAATATCGGCATTTAGCCACAGCACC-3′; D280N, 5′-ATCAGACCATACAATTTCAACAACTGG-3′ and 5′-CCAGTTGTTGAAATTGTATGGTCTGAT-3′; and H389A, 5′-GGCTTACCAAACTATTTGGTTTATGCTATAGAGGAAGAGAACCATTGATGGATCC-3′ and 5′-GGATCCATCAATGGTTCTCTTCCTCTATAGCATAAACCAAATAGTTTGGTAAGCC-3′.

*Escherichia coli* BL21(DE3) pLysS cells were transformed with pNIFTY/MBP containing the gene of interest. A fresh overnight culture of *E. coli* BL21 transformants was diluted 1 : 50 in autoinduction medium [39] containing 100 µg ml^−1^ carbenicillin and grown at 18°C for 24 h. Cells were harvested by centrifugation at 3300*g* for 30 min at 4°C, and the pellet was resuspended in lysis buffer (25 mM Tris–HCl, pH 7.5, 250 mM NaCl, 30 mM imidazole) and kept frozen at −80°C until lysis.

### 5.2. Cell lysis and protein purification

Resuspended cells were supplemented with lysozyme and DNase I (Sigma-Aldrich), and lysed on a constant cell disruptor system at 30 kpsi (Avestin). The lysate was spun down for 30 min at 31 000*g* and 4°C. The soluble fraction was passed through a 0.2 μm filter and bound to Ni^2+^-charged IMAC resin (GE Healthcare) by gravity flow. Unspecific proteins were washed off by applying 10 column volumes (CVs) of lysis buffer. The protein of interest was eluted with 3 CV lysis buffer supplemented with 200 mM imidazole. The eluate was dialysed against buffer A (25 mM Tris–HCl, pH 7.5) for 2 h at room temperature (RT). The tag was cleaved off by adding 500 μg TEV protease and incubation for 16 h at 4°C. Cleaved protein was injected onto a 5 ml HiTRAP Q FF (GE Healthcare) equilibrated in buffer A. The MBP tag was removed by washing with 2 CV buffer A containing 150 mM NaCl. *Sc*Mnn9 or *Sc*Van1 was eluted using 3 CV buffer A containing 400 mM NaCl. Fractions containing protein of interest were pooled and dialysed against buffer B (25 mM Tris–HCl, pH 7.5, 150 mM NaCl and 2 mM MnCl_2_) for 2 h at RT. The sample was concentrated to 1 ml and injected onto a Superdex 75 size-exclusion column equilibrated in buffer B. Fractions containing *Sc*Mnn9 or *Sc*Van1 were pooled, concentrated to 5 mg ml^−1^, flash frozen in liquid nitrogen and stored at −80°C.

### 5.3. Protein crystallization, data collection and refinement

Octahedral *Sc*Mnn9 crystals were grown by vapour diffusion in 1 µl sitting drops containing 0.5 µl protein and 0.5 µl mother liquor (0.1 M HEPES, pH 7.5 and 2 M ammonium sulfate). Crystals were transferred to 50% Na–malonate, pH 7.5, containing mersalyl acid and soaked for 16 h at 20°C. Soaked crystals were directly frozen in liquid nitrogen, because the Na–malonate acted as cryoprotectant [40]. A 38-fold redundant 2.2 Å dataset collected at beamline ID14-4 at the European Synchrotron Radiation Facility (ESRF, Grenoble, France) was used for SAD. Initial phases were calculated from a single Hg site using the SHELX program suite [41]. Solvent flattening was also performed with SHELX, which gave a good-quality map showing protein/solvent boundaries and some secondary structure elements. The map was used as input for warpNtrace [42], which built 200 of 305 residues with one molecule in the asymmetric unit. Model building and refinement was continued in COOT [43] and REFMAC [44], yielding the final model with statistics shown in table 1.

Crystals of *Sc*Mnn9-D236N were transferred to 50% Na–malonate, pH 7.5 and soaked with 3 mM GDP and 10 mM MnCl_2_ for 10 min at 20°C and then flash frozen in liquid nitrogen. Diffraction data were collected at beamline ID14-4 at the ESRF to 2.0 Å. Refinement was initiated from the native structure. This revealed well-defined |*F*_O_| − |*F*_C_| electron density for GDP and Mn^2+^. Model building and refinement was continued as described above and resulted in the statistics of the final model as shown in table 1.

### 5.4. Coupled fluorimetric *Sc*Mnn9 enzyme assay

To measure enzyme kinetics 500 nM *Sc*Mnn9 was incubated in 20 mM HEPES, pH 7.5, with 10 mM MnCl_2_, 0.2 mg ml^−1^ BSA and 10 µM of the α-1,6-specific mannosidase Aman6 [29]. Initial rates of mannotriose formation were measured with substrate concentrations ranging from 0 to 1.2 mM GDP-Man and 10 mM 4-methylumbelliferyl-α-d-mannopyranoside. Initial rates of mannosyltransfer were measured with concentrations ranging from 0 to 10 mM 4MU-Man and 1.2 mM GDP-Man. Product formation was determined fluorimetrically at *λ*_ex_ = 360 nm and *λ*_em_ = 460 nm.

### 5.5. Binding kinetics of *Sc*Mnn9 and *Sc*Van1

The *K*_d_ of *Sc*Mnn9 and *Sc*Van1 was determined on an Octet RED384 (Fortebio). *Sc*Mnn9 was biotinylated with EZ-link NHS-PEG4-Biotin (Thermo Scientific) according to the manufacturer's instructions, and the activity of *Sc*Mnn9 was confirmed by FACE. *Sc*Mnn9 was bound at 25 µg ml^−1^ to streptavidin sensors in buffer C (100 mM HEPES, pH 7.5, 5 mM MnCl_2_, 1 mg ml^−1^ BSA, 0.015% BRIJ 35). The loaded sensors were incubated with *Sc*Van1 at concentrations ranging from 0 to 15 µM in buffer C. Association was measured over 10 min and dissociation in buffer C was measured over 20 min. The measurements were performed in triplicate and full kinetic fit was used to determine kinetics.

### 5.6. *In vitro* mannosyltransferase assay

Mannosyltransferase assays contained 500 nM *Sc*Mnn9 and/or 500 nM *Sc*Van1, 20 mM HEPES, pH 7.5, 10 mM MnCl_2_, 10 mM α-1,6-d-mannobiose and 1.2 mM GDP-Man. The reaction was incubated for 16 h at 30°C and stopped by adding three volumes of ice-cold ethanol. The reaction products were labelled with 750 nmol ANTS and used in a FACE, as described elsewhere [45]. For the reaction containing mannose as acceptor substrate, reactions were as described above, except for 1 mM mannose and 20 mM GDP-Man as acceptor and donor, respectively. The products were digested with α-1,6-mannosidase (NEB) according to the manufacturer's instructions.

### 5.7. Transformation of *Saccharomyces cerevisiae* with MNN9 and *in vivo* assays

*Saccharomyces cerevisiae* BY4741 wild-type (MAT a; *his3*Δ* 1; leu2*Δ* 0; met15*Δ* 0; ura3*Δ* 0*) and *ΔMNN9* (Mat a; *his3D1; leu2D0; met15D0; ura3D0; YPL050c::kanMX4*) were obtained from EUROSCARF. The yeast expression plasmid pRS315 was a kind gift of Michael Stark (University of Dundee). The gene of *MNN9* including the 5′- and 3′-untranslated regions, according to the yeast promoter atlas, was cloned into pRS315 using the oligonucleotides 5′-AAA**GGATCC**ATCACAGAACCGGAAAATAGTAGCCAC-3′ and 5′-TTT**GAGCTC**CTCAAGCTCAGAAATTAGTTGTTGTAGC-3′ (restriction sites for *Bam*HI and *Sac*I shown in bold, respectively). A single colony of *S. cerevisiae* was used to inoculate 20 ml YPD (1% (w/v) yeast extract, 2% (w/v) peptone, 2% (w/v) glucose) medium and the cells were grown for 16 h at 30°C and 220 r.p.m. The culture was spun down for 5 min at 1000*g*, and the pellet was washed twice in 5 ml sterile ddH_2_O. For transformation, 100 µl of resuspended cells were mixed with 2 µg pRS315 [37] containing the gene of interest and 300 µl of transformation buffer (40% PEG 3350, 120 mM lithium acetate, 0.8 mg ml^−1^ single-stranded salmon sperm DNA). The transformation mixture was incubated at 42°C for 45 min. Cells were spun down for 5 min at 6000*g*, the supernatant was aspirated and the pellet was resuspended in 100 µl sterile ddH_2_O. The transformed cells were spread on DO-agar plates (0.7% (w/v) yeast nitrogen base, 2% (w/v) glucose, 0.07% (w/v) adenine, complete supplement mixture DCS0099 (Formedium), 2% (w/v) agar) lacking leucine and incubated for 2 days at 30°C. Single colonies of transformed cells were used to inoculate 1 ml YPD medium, and the culture was left to grow for 8 h at 30°C. Growth curves were determined measuring the absorption at 600 nm, starting by diluting cells to OD_600_ = 0.05 in YPD followed by growth for 12 h at 30°C under agitation. Mannosylation defects were determined by diluting *S. cerevisiae* to OD_600_ = 0.2, making 1 : 10 serial dilutions and spotting 2 µl of cells onto YPD plates containing either no supplement, sodium orthovanadate (2.5 mM, Sigma-Aldrich) or hygromycin B (25 and 50 µg ml^−1^, Sigma-Aldrich). Cells were grown for 2 days at 30°C.

### 5.8. Invertase assay

Detection of mannosylation defects in *S. cerevisiae* transformants was carried out as described by Ballou [46]. Briefly, *S. cerevisiae* cells were grown to mid-log phase (OD_600_ ∼ 0.35) in YPD at 30°C and 220 r.p.m. The medium was changed to YPD containing only 0.05% glucose, and cells were grown for 3 h at RT. Cell pellets were washed with 20 mM sodium azide and resuspended in TBS + 1 mM PMSF. Cells were lysed with 0.45 µm glass beads four times by vortexing vigorously for 30 s, keeping the cells on ice for 2 min between each lysis step. The samples were run over an 8% polyacrylamide gel for 2.5 h at 20 mA at RT. The gel was incubated for 10 min in an ice-cold 0.1 M sucrose solution in 0.1 M sodium acetate, followed by incubation in the same solution at 37°C. The gel was rinsed twice with water, and a solution of 2,3,5-triphenyltetrazolium in 0.5 M NaOH was added and heated until staining was sufficient. The same samples were run again, and the gel was stained in Coomassie brilliant blue for loading control.

## Supplementary Material

Supplementary material

## Data accessibility

The coordinates and structure factors for ScMnn9-D236N + Mn^2+^ + GDP have been deposited with the PDBe and can be accessed under entry code 3ZF8.
