# Supplementary material for: Yeast Mnn9 is both a priming glycosyltransferase and an allosteric activator of mannan biosynthesis
Source: Open Biol. 2013 Sep;3(9):130022. doi: 10.1098/rsob.130022 (PMC3787745; doi:10.1098/rsob.130022)

Yeast Mnn9 is both a priming glycosyltransferase and an allosteric activator of mannan biosynthesis

Alexander Striebeck, David A. Robinson, Alexander W. Schuettelkopf and Daan M. F. van Aalten

Supplementrary Figure S1

PAGE gel of invertase of native and complemented S. cerevisiae Δ*MNN9* strains

An 8 % PAGE gel was loaded with cell lysate from the native and complemented S. cerevisia transformants indicated. The gel was stained with Coomassie brilliant blue to serve as loading control for the invertase assay gel shown in Fig. 6b.

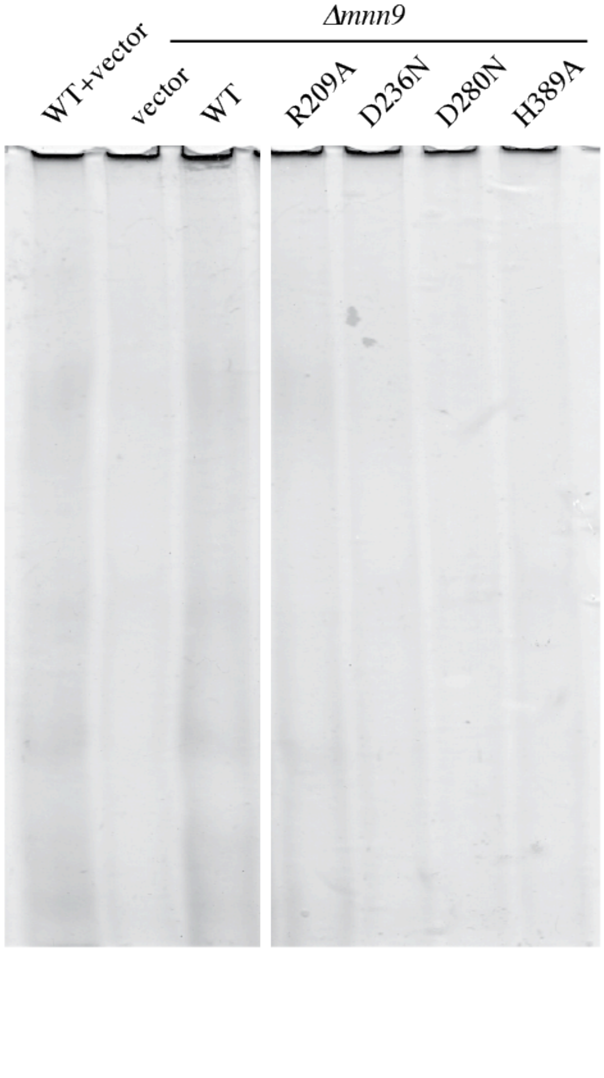

Supplement: Supplementary material [file rsob130022supp1.pdf]
